# ABRF Core Administrators Network: The Early Years

**Published:** 2026-03-11

**Authors:** Philip Hockberger, Julie Auger, Paula Turpen, Susan Meyn

**Affiliations:** 1 Office for Research, Northwestern University, Evanston, IL USA (retired); 2 Research Operations Salk Institute for Biological Studies, La Jolla, CA USA; 3 Research Resources University of Nebraska Medical Center, Omaha, NE USA (retired); 4 Office for Research Vanderbilt University Medical Center, Nashville, TN USA

**Keywords:** core administration, shared research resources, compliance, federal policy, regulations, NIH, NCRR, OMB

## Abstract

This article presents the authors’ perspectives on the origins and early impact of the Association of Biomolecular Facilities (ABRF) Core Administrators Network (CAN). Formed in 2010 amid the rapid expansion of core facilities across U.S. universities and medical centers, CAN addressed growing demand for guidance on compliant service pricing and cost-recovery practices. Through early leadership and targeted educational programs, CAN helped resolve policy and regulatory questions, raised awareness among federal agencies and institutions of the central role of core facilities in modern research, and advocated for sustained investment to preserve their effectiveness and efficiency. In partnership with ABRF, CAN drove membership growth, positioned ABRF as the national organization for core administrators, and catalyzed the creation of institutional sponsorships These foundational efforts established CAN as a national leader in core administration and strengthened the standing of core facilities as essential assets for research instututions.

## Introduction

On July 14–15, 2009, the National Center for Research Resources (NCRR) of the National Institutes of Health (NIH) convened a public workshop in Bethesda, MD.^1^ The goal was to address the rapid growth of biomedical research core facilities 1 at US universities, medical centers, and research institutes and to share guidance for managing these resources efficiently and in compliance with federal requirements. Organized by Greg Farber, Director of the Office of Extramural Activities at NCRR, and Louise Ramm, NCRR Deputy Director, the workshop drew representatives from multiple federal research agencies as well as stakeholders from universities and organizations operating core facilities. Three of us (JA, PT, SM) attended. Participants assessed NIH funding mechanisms for core facilities, identified common operational challenges, and discussed strategies to improve access, administrative management, training, utilization, and quality assurance.

The workshop galvanized the core facility community. A prominent theme was recognition of the rapid expansion of fee-for-service models at universities and medical centers and the corresponding need for clear guidance to ensure compliance with federal cost-accounting policies. Existing policies from the US Office of Management and Budget (OMB)—particularly Circular A-21—were judged to provide limited direction. Although A-21 (and, since 2014, its successor, the Uniform Guidance at 2 CFR 200.468) addresses “specialized service centers” (for example, animal care and computer centers), these provisions cover only a subset of academic research cores operating on a service-fee basis. Workshop participants agreed that this policy gap required prompt attention.

NIH subsequently led efforts to address this gap. Beginning in 2010, it established a task force to develop comprehensive guidance for costing core facilities (discussed in the following section). In 2011, Greg Farber and Linda Weiss (Director of the National Cancer Institute’s Office of Cancer Centers) published a policy commentary highlighting the value of cores and the need for clear cost-recovery principles, building on the workshop’s findings and foundational principles.^2^ Their article helped elevate national awareness of the importance of core facilities and set the stage for the ensuing 15 years of progress in core administration. As discussed in the following section, the ABRF also played a pivotal role in raising the visibility and leadership of biomedical core facilities in partnership with public and private research institutions.

## Creation of the Core Administrators Network

The idea for the Core Administrators Network (CAN) originated with Tony Yeung and Michelle Detwiler, former presidents of the ABRF, which promotes education and career advancement for scientists working in biomedical research core facilities. Recognizing the growing importance of core facilities in the research ecosystem, challenges identified at the NIH-NCRR workshop, and the need to engage institutional leaders, Detwiler invited two of us (SM and PT) at the ABRF 2010 Annual Meeting to lead a new committee focused on core facility administration, and we agreed.

On September 22, 2010, Yeung and Detwiler convened a conference call with several university administrators overseeing core facilities (including coauthor PH) to discuss institutional challenges and priorities and to clarify how ABRF could help facilitate solutions. Participants expressed unanimous interest in collaborating, and monthly meetings were initiated. These early meetings led to the formation of a formal ABRF committee charged with developing a national network of core administrators. Initially named the Core Administrators Network Coordinating Committee (CAN-CC), the group later became known simply as CAN. Susan Meyn (Vanderbilt University Medical Center) and Paula Turpen (University of Nebraska Medical Center) were selected as cochairs. Other committee members included Phil Hockberger (Northwestern University), Diane Tabarini (Memorial Sloan Kettering Cancer Center) and Connie Nicklin (University of Florida). Julie Auger (University of California, San Francisco) joined in 2011, and Karen Jonscher (University of Colorado, Denver) served as the first liaison to the ABRF Executive Board.

CAN held its first official meeting on November 4, 2010, and drafted a mission statement and goals for 2011. Early priorities included the following: publicizing and expanding the network; creating opportunities for administrators to discuss shared interests and challenges; and facilitating networking at ABRF annual meetings. Subsequent meetings, informed by an ABRF membership survey, added the following goals: (1) developing a database of core administrators; (2) identifying and describing core management models; (3) collecting and disseminating tools for core administration; and (4) collaborating with the ABRF 2011 Planning Committee to provide sessions for core administrators. CAN quickly organized a ‘Meet & Greet’ networking event and two workshops: Institutional Core Management (led by coauthors PT & SM) and Core Labs as Businesses (led by Nick Ambulos, University of Maryland School of Medicine, & Steve Bobin, Dartmouth Medical School). In addition, the committee sponsored a presentation by Mark Lively (Wake Forest University School of Medicine), a past president of both ABRF and Federation of American Societies for Experimental Biology (FASEB) and a member of the NIH Council of Councils, on the planned dissolution of the NCRR directorate in December 2011^2^.

The dissolution of NCRR was a significant setback for the core facility community, as NCRR had been the primary advocate for NIH-wide support of core facilities.^3^ Immediately after the 2011 meeting, CAN and the ABRF Executive Board organized meetings with leaders at the NIH 3 and the National Science Foundation (NSF) to explore ways to strengthen communication and collaboration among federal agencies, ABRF members, and their institutions. The response was enthusiastic, marking the beginning of a new partnership between the core facility community and federal agencies, with ABRF and CAN playing central roles.

## Early Influence of CAN on Core Administration

In 2012, CAN presented a poster at the ABRF Annual Meeting in Orlando, FL, summarizing a survey of core administrators (Appendix 1 shows an image of the poster). The survey captured job descriptions and responsibilities, institutional types, oversight models, and funding sources for core facilities. CAN also hosted an interactive roundtable, Models for Core Management and Administration: Perspectives from members for the Core Administrators Network. These well-attended activities led to three outcomes with broad and lasting impact:

1. Community building via listserv
  CAN launched a listserv in 2012 to enable administrators to pose questions, share ideas, and exchange best practices. Early discussions helped the committee surface emerging and persistent challenges, and fostered a collaborative community committed to problem-solving. The listserv not only accelerated dissemination of best practices, it also built an enduring sense of camaraderie among administrators.
2. Establishment of an administrative track at ABRF
  CAN organized a dedicated administrative track for the 2013 ABRF Annual Meeting, with sessions designed specifically for core administrators. Topics were solicited from the administrator network to ensure relevance and alignment with common needs. Previously, administrative content was dispersed among research group talks, satellite workshops, or plenaries. The success of the 2013 program led to the administrative track becoming a permanent feature of ABRF meetings, with CAN taking primary responsibility for its development (Appendix 2 lists 2013–2016 topics).
3. Contribution to national cost-accounting guidance
  In 2010, two CAN leaders (coauthors JA and SM) were invited by Joe Ellis (NIH) to join a task force addressing cost-accounting issues raised at the 2009 NIH-NCRR Workshop. Working in parallel with the formation of CAN, the task force addressed frequently asked questions on cost accounting for core facilities. CAN provided iterative feedback, many elements of which were incorporated into the final NIH document, *FAQs for Costing of NIH-Funded Core Facilities* (NOT-OD-13-053, published April 8, 2013). The guidance helped research-intensive institutions operate core facilities in compliance with federal cost-accounting principles.

In 2013, CAN coauthored a Journal of Biomolecular Techniques (JBT) article on best practices for managing external customers of core facilities — the first JBT paper focused on core administration.^4^ The article outlined institutional policies (best practices) and procedures (terms and conditions) that enable external use, summarized applicable federal regulations, and provided practical guidelines. The authors received the JBT Outstanding Manuscript Award at the ABRF 2014 Annual Meeting.

In 2014, CAN contributed to ABRF’s response to the NIH Request for Information (RFI) on the Shared Instrumentation Grant Program, NOT-OD-14-104. The response^5^ advocated for placing instruments in core facilities to optimize access, operations, utilization, and sustainability, and recommended increasing Small Instrumentation Grant (SIG) awards to $750,000 to bridge the gap between SIG and High-End Instrumentation (HEI) awards. Subsequent SIG program changes reflected these recommendations, underscoring ABRF and CAN’s growing influence on NIH policies affecting core facilities.

In 2015, CAN worked with the ABRF Executive Board to organize a joint ABRF-NIH Workshop at the St. Louis Annual Meeting focused on practices that enhance core facility efficiency. NIH leaders outlined support mechanisms and policies, while institutional leaders discussed barriers and solutions associated with centralized core oversight. Videos, slides, and a Workshop Report are available online.^6–9^ The workshop established ABRF and CAN as key conduits between institutions and federal policymakers.

In 2016, CAN coauthored an article^10^ describing approaches to evaluate core performance across 8 domains: general management; research and technical staff; financial management; customer base and satisfaction; resource management; communications; institutional impact; and strategic planning. The paper highlighted lessons learned to improve effectiveness and efficiency and argued that systematic performance evaluation elevates the institutional visibility of cores, improves staff satisfaction, and strengthens long-term sustainability.

Also in 2016, CAN coordinated input from the administrators network for ABRF’s response to the NIH-National Institute of General Medical Sciences (NIGMS) RFI on the Need for and Support of Research Resources for the Biomedical Research Community, NOT-GM-16-103. Recommendations included the following: adapting grant structures (requirements, reporting, authorship) to recognize the distinct contributions of core scientists; strengthening the role of NIGMS in promoting shared resource use by principal investigators (PIs), especially in large program projects; and expanding funding opportunities to support shared research resource personnel and operations.

## Professional Development of Core Administrators

Participation in CAN membership and events became a key pathway for core administrators to develop professional skills. To broaden representation across the ABRF membership, the committee expanded outreach at ABRF national and chapter meetings, instituted a formal process for selecting new members, and adopted three-year rotating terms to ensure new voices are heard and to support professional growth (see Appendix 3 for names and institutions of CAN membership from 2010–2016).

One of the most gratifying aspects of CAN participation was the opportunity for members to assume leadership roles within ABRF. In 2013, Paula Turpen (University of Nebraska Medical Center) and Bill Hendrickson (University of Illinois, Chicago) were elected to the ABRF Executive Board, paving the way for subsequent administrators, including Julie Auger (University of California, San Francisco) and Andy Chitty (Oregon Health Sciences University) in 2016; Nancy Fisher (University of North Carolina) and Sheenah Mische (New York University Langone Medical Center) in 2017; and Claudius Mundoma (University of Colorado, Boulder) in 2018. Two core administrators later served as President (Hendrickson 2014-2016; Chitty 2018-2019) and two as Treasurer (Turpen 2014-2016; Auger 2017-2019). CAN members also contributed in other ABRF leadership roles, such as chairing the Annual Meeting Planning Committee (Hockberger 2014; Meyn 2017).

## Conclusion

Now in its 16^th^ year, CAN continues to provide leadership and expertise in core facility administration. Still, sustained effort is needed to ensure long-term viability of shared research resources, as highlighted in several FASEB reports^11^ and a related publication.^3^ Looking back, we are gratified to have supported the early growth and development of CAN, and we look forward to future core administrators maintaining this momentum and building upon these early successes.

### Financial Support / Conflict of Interest Statement

The authors acknowledge that no financial support was used to generate this report and that its contents pose no conflict of interest.

## Footnotes

1 Core Facilities are shared research resources that provide specialized instruments, services, and expertise.

2 The dissolution of the NCRR resulted in transfer of its programs to other Institutes & Centers within NIH. The SIG/HEI grant programs were initially transferred to the Office of the Director under the Division of Program Coordination, Planning, and Strategic Initiatives (DPCPSI) and later moved to the Office of Research Infrastructure Programs (ORIP).

3 The EB held a conference call with NIH-DPCPSI leaders on July 27, 2011, to share concerns regarding the dissolution of NCRR and how to ensure that core facilities remain a priority of the NIH. This led to the invitation to NIH leaders to attend the ABRF ’12 meeting, which they accepted, and to share their vision and continued support of core facilities with the ABRF community.

## Appendices

### Appendix 2. Core Administrator Track topics at ABRF meetings, 2013-2016

2013

- Research Compliance: What Core Directors Need to Know
- Career Opportunities for Core Directors
- Regional Resource Sharing/Collaboration/Consolidation: Models of Effective Resource Access and Utilization
- Metrics of Success for Core Facilities
- Core Facility Financial Essentials
- Embracing Lab Biocontainment & Biosafety: From Policy to Best Practice
- Strategies for Managing Multi-Sponsored Core Facilities

2014

- Career Paths in Core Facilities: Overcoming HR & Compensation Obstacles/Recruitment & Retention of Core Staff
- Crucial Accountability: Tools for Resolving Broken Promises and Violated Expectations
- Business Planning, Forecasting and Marketing without a Crystal Ball
- Internal and External Review of Core Facilities
- Visualization Tools for Core Facility Administrators
- Big Data Panel
- External Customers and Regional Resource Sharing
- Setting Service Rates: Institutional

2015

- Intercore Partnerships: Enhancing Research Support
- Federal Compliance and Considerations for Development of Research Services Cost Models and Rate Calculations
- Discussion on Establishing Federally Compliant Service Units
- Lean Enterprise Practices to the Operations of Cores
- Building a Sustainable Portfolio of Core Facilities: Part 1: A Case Study
- Building a Sustainable Portfolio of Core Facilities: Part 2: Challenges & Opportunities
- Trending Core Business Challenges and How to Overcome Them

2016

- Reproducibility in Science: It’s Everyone’s Problem
- Lean Management in Cores: Specific Tools to Help with Core Productivity and Client Satisfaction
- Professional Development: Promoting Core-Specific Job Families
- Career Development: Mentoring Models
- Evolving Core Technologies
- Business Skills: Customer Management in a Shared Resource Environment
- Lessons Learned: How to Choose a Centralized Billing System that Best Suits your Institution
- Improving Openness and Reproducibility of Scientific Research
- NIH and Federal Policy Update

### Appendix 3. CAN membership in the early years

**Table:**

2010-⁠2013	**Paula Turpen (co-chair), University of Nebraska Medical Center; Susan Meyn (co-chair), Vanderbilt University Medical Center;** Phil Hockberger, Northwestern University; Connie Nicklin, University of Florida ICBR; Diane Tabarini, Memorial Sloan Kettering Cancer Center; Julie Auger, University of California, San Francisco; Karen Jonscher (EB liaison), University of Colorado-Denver
2013-⁠2016	**Julie Auger, University of California, San Francisco (co-chair); Phil Hockberger, Northwestern University (co-chair);** Connie Nicklin, University of Florida ICBR; Diane Tabarini, Memorial Sloan Kettering Cancer Center; Susan Meyn, Vanderbilt University Medical Center; Veronica Rice, Nationwide Children’s Hospital; Amy Wilkerson, Rockefeller U.; Andrew Vinard, U. Miami Medical School; and Susanna Perkins, U. Mass Medical School; George Grills (EB liaison), Cornell University
